# Societal factors influencing the implementation of AI-driven technologies in (smart) hospitals

**DOI:** 10.1371/journal.pone.0325718

**Published:** 2025-06-12

**Authors:** Ysanne de Graaf, Anam Ahmed, Carmen Sanges, Laura Herbst, Hubertus J.M. Vrijhoef

**Affiliations:** 1 Panaxea, Den Bosch, The Netherlands; 2 Maastricht University, Maastricht, The Netherlands; 3 University Hospital of Würzburg, Würzburg, Germany,; 4 Fraunhofer Institute for Production Technology IPT, Aachen, Germany; Universiti Teknologi Malaysia - Main Campus Skudai: Universiti Teknologi Malaysia, MALAYSIA

## Abstract

**Introduction:**

The introduction of AI in healthcare promises benefits, but also faces challenges. Currently, one of these challenges is the lack of information on the societal aspects of implementing AI in healthcare. This study aims to: 1) identify which societal factors play a key role in the implementation of AI-driven technology in (smart) hospitals according to different stakeholder groups; 2) examine how these factors play a role within (smart) hospitals by discussing their facilitators, barriers, possibilities, and preconditions; and 3) develop a societal guide to serve as a roadmap for an implementation process of AI in a healthcare setting.

**Methods:**

A survey was conducted, followed by four focus group interviews (FGIs). In the survey, participants (n = 7) assessed the relevance of factors for inclusion in the FGIs using a rating scale from 1 to 5 (1 = irrelevant, 5 = relevant). In each FGI, 2–3 participants discussed how these societal factors play a role in the implementation of AI technology in (smart) hospitals. By combining and categorizing these insights, a societal guide was set up to provide a structured approach for implementation of AI-driven healthcare innovation.

**Results:**

The survey revealed that 9 out of 10 proposed factors were considered relevant (90%). The FGIs demonstrated uncertainty surrounding the (future) use of AI technologies within (smart) hospitals. As this field is still in its early stages, there are limited established methodologies and (regulatory and ethical) frameworks for implementation. While much knowledge exists on different factors concerning AI in (smart) hospitals, this knowledge is often siloed. This knowledge must be integrated across stakeholders to adequately prepare for the deployment of AI technologies. The societal guide developed addresses ethical and regulatory considerations, while also covering important human-centred factors for AI implementation in healthcare.

**Conclusion:**

Engaging various stakeholders throughout different phases of AI implementation in (smart) hospitals (i.e., development, implementation, monitoring and evaluation phase) is key for fostering a collaborative approach. Recognizing the interdependence and collective impact of factors is essential for creating a successful implementation trajectory.

## Introduction

Advanced technologies are reshaping medical services, leading to the emergence of ‘smart’ healthcare. Smart healthcare involves integrating information and communication technology (ICT) with traditional hospital and doctor-centred healthcare systems, with the aim of enhancing and personalizing healthcare services [1]. Smart hospitals are at the forefront of this development, using data and technology to offer a wide range of services for patients, medical staff and administrators. These types of hospitals rely on data-driven insights, including machine learning models and artificial intelligence (AI)-powered medical devices, to accelerate, augment or simplify the work of hospital staff. Smart hospitals have been shown to improve (preventive) patient care and the efficiency of healthcare processes [2]. Recent research emphasizes that smart hospitals also promote better decision-making by providing real-time information and predictive analytics, which lead to more personalized and timely treatment [3].

AI is an important component of smart healthcare and is increasingly applied in various fields, from predictive analytics to medicine manufacturing to population health management [4]. AI offers significant potential, such as enhancing diagnostic accuracy, reducing administrative burdens, and saving both time and costs [5–9]. However, this potential comes with considerable risks. AI can compromise patient data privacy, generate biased outcomes due to algorithmic bias, and exacerbate existing health inequalities [5,7–12]. Despite its potential, the actual uptake of AI in healthcare has progressed slower than anticipated [13,14]. Contributing factors include high implementation costs, limited compatibility with existing hospital infrastructures, and concerns around the accountability and transparency of AI-driven systems [13,15].

Additionally, there is still a limited understanding of the specific needs and considerations surrounding the implementation of AI in healthcare. Effective and responsible implementation of AI presents numerous challenges, also due to the current lack of information on the societal impact of AI in healthcare. Societal impact refers to the effects -both positive and negative- that a project, activity, programme, or policy has on individuals and communities [16]. Healthcare is inherently a social institution, and AI’s introduction into this space affects a wide range of stakeholders, from patients and healthcare professionals (HCPs) to hospital managers and policymakers. Therefore, research on the societal implications of AI in healthcare provides the foundation for developing strategies that can maximize the benefits of AI, while mitigating its risks. By addressing these societal factors, we can better ensure that AI deployment is both ethical and effective in improving healthcare outcomes. Societal perception of AI in healthcare varies, with some individuals open to AI for health purposes, while many still prefer human practitioners for complex issues [4,9]. This variability in acceptance highlights the importance of understanding key factors for successful implementation. Previous studies have identified several critical factors for the implementation of smart healthcare systems, and examined the variable attitudes of healthcare personnel, patients, and the general public towards AI in healthcare [9,17]. Despite these insights, a significant gap remains in our understanding of how to ethically and practically integrate AI into real-world healthcare settings. Further complicating this is the challenge of aligning the rapid pace of technological innovation with the slower pace of regulatory and policy adaptation, which has been identified as a key barrier to successful AI adoption [15,18].

To address these multi-level challenges, there is a need for a societal guide that supports the implementation of AI-driven innovation in healthcare. This guide should offer a framework for the responsible use of AI in (smart) healthcare settings, taking into account social, ethical, and regulatory aspects. Recent research emphasized the importance of incorporating mechanisms for continuous monitoring and evaluation to ensure that AI systems remain aligned with changing patient needs and societal values [19,20]. To be effective, the guide should reflect the collective insights and perspectives of diverse stakeholder groups, including HCPs, hospital implementation managers, researchers, technology developers, and policymakers.

Therefore, the objectives of this study are to: 1) identify which societal factors play a key role in the implementation of AI-driven technology in (smart) hospitals according to different stakeholder groups; 2) examine how these factors play a role within smart hospitals by discussing their facilitators, barriers, possibilities, and preconditions; and 3) develop a societal guide to serve as a roadmap for the implementation process.

## Methods

### Study setting

This study is part of the EU Horizon2020 project AIDPATH (grant agreement number 101016909) [21,22]. AIDPATH aims to develop an AI-driven, automated chimeric antigen receptor T cell (CAR-T) manufacturing platform at the point of care as a treatment for acute leukaemia and lymphoma. The AIDPATH project seeks to automate the manufacturing process using AI to optimize resource management and contribute to cost-effective platforms, showcasing the potential of AI in healthcare settings, including smart hospitals [23–25].

To meet the study objectives a survey was conducted followed by focus group interviews (FGIs).

In the survey and FGIs, the AIDPATH project was used as a case study, with a focus on AI-driven manufacturing as the primary example to explore and generalize the societal factors crucial for implementing AI-driven healthcare innovation.

### Survey

To develop the topic guide for FGIs, relevant factors related to the societal aspects of implementing AI technologies in (smart) hospitals were identified, and the relevance of these factors was validated through a survey.

#### Survey topics.

Potential topics for a topic guide for FGIs were derived from a review conducted by Sony et al. (2023) [17], which originally focused on Health 4.0. The identified factors were then adapted to better align with the context of our research, which is centred around implementation of AI technologies in (smart) hospital settings. Prior to inclusion of the factors in the topic guide for the FGIs, these factors were validated on their relevance beforehand. To achieve this, a survey in English language was distributed among all seven work package leaders within the AIDPATH-consortium. The survey contained 10 societal factors related to the implementation of AI technologies in smart hospitals, namely:

1. Digital integration and interconnectedness of the healthcare ecosystem;
2. Utilization of big data and analytics;
3. Management of digital healthcare supply chains;
4. Strategies for promoting the use of AI technologies in medicine;
5. Promotion of a culture for the use of AI technologies in medicine;
6. Leadership in healthcare innovation;
7. Development of skills among healthcare employees;
8. Adoption of new business models;
9. Regulatory aspects of AI technologies in medicine;
10. Ethical aspects of AI technologies in medicine.

Please see S1 File for the survey questions.

#### Data collection and analysis.

Participants were asked to assess the relevance of each factor using a rating scale from 1 to 5, where 1 signified irrelevance and 5 signified relevance. Throughout the survey, ‘relevant’ meant bearing significance for the societal aspects for implementing AI technologies in smart hospitals, while ‘irrelevant’ meant bearing no significance. The ‘N/A’ option was available as well for each factor. For each factor, it was possible to provide additional comments and/or suggestions related to their assessment. Furthermore, participants had the opportunity to suggest any additional factors that are relevant for the successful implementation of AI technologies in smart hospitals.

The survey was distributed to the work package leaders via email, which contained a weblink to the survey on the Survey Monkey® platform in September 2023. All responses were recorded anonymously, and participants had a right to withdraw from the study at any time. The survey data were collected in October 2023. Survey data analysis was performed in Microsoft Excel. Percentage of experts indicating a particular level of relevance on the 1–5 scale were calculated for each factor. Factors were included in the topic guide if at least 75% of the participants found the factors relevant. Factors between the scores 1–3 were considered to be of low relevance. Factors between the scores 4–5 were considered to be of high relevance. The open-question responses were taken into account when interpreting the numerical data.

### Focus group interviews

FGIs were held to discuss how the societal factors played a role in the implementation of AI technology in smart hospitals.

#### Sampling.

Participants were selected and sampled using purposive sampling methods, via the professional networks of the AIDPATH consortium members. Participants were selected based on their active involvement in the development and/or implementation of AI-related initiatives in healthcare. Although no strict minimum years of experience were required, most had several years of relevant expertise, as evidenced by their roles and responsibilities. Familiarity with the topic was assessed through their demonstrated involvement in AI development or implementation within their organizations. Maximum variation within the stakeholder groups was aimed to be achieved by including individuals from various professional backgrounds (e.g., HCPs, policymakers, technology developers, researchers)and level of familiarity with ethical or social aspects of AI [26]. Invitations to participate in a FGI were extended to participants via email. Participants were recruited in the period October 2023 till December 2023.

#### Data collection and analysis.

The FGIs followed a topic guide, compiled based on the factors identified from the survey. The FGIs started with an introductory round, a brief overview and explanation of the study objectives, followed by the main questions, and a closing. The main questions covered the societal factors related to the implementation of AI-technologies in (smart) hospitals. Per factor, questions were set up focusing on the possible related facilitators, concerns/barriers, possibilities, and preconditions (please see S2 File for the topic guide). Participants were provided with the opportunity to share their own insights or experiences, ask questions, and react to other perspectives. ‘Participatory learning and action’ techniques were employed to facilitate equal stakeholder input, promoting active engagement among participants [27]. All FGIs were led by a researcher (YdG or AA) and were conducted in the presence of at least one other researcher (YdG or AA). The FGIs took place online via Microsoft Teams in January and February 2024 and lasted 60 minutes on average. The FGIs were recorded after obtaining consent from the participants. The recordings were transcribed verbatim using the Amberscript software. The responses of participants were thematically coded and analysed by two researchers independently. The researchers (YdG and AA) assigned a code to text segments that relate to specific themes and then compared these codes. Data were analysed both descriptively (summarizing the main characteristics of the data) and exploratively (search for more complex patterns, relationships, or insights within the data).

### Societal guide

Based on the insights and considerations gathered from various stakeholder groups during the FGIs, a societal guide was developed. This was done by combining stakeholder insights and sorting them into three phases, each corresponding to different stages of implementation: development, implementation, and monitoring and evaluation thereafter. Factors included in the guide have been further categorized into three levels: micro/individual, meso/organizational and macro/system-level. By organizing the factors across these three levels, the guide provides a holistic approach to AI implementation, ensuring that the perspectives of individuals, organizations, and the broader healthcare system are all taken into account. The societal guide provides a structured roadmap for the implementation of AI-driven healthcare innovation in (smart) hospitals. This guide is designed to address key societal factors that influence the implementation of AI technologies and to offer a comprehensive framework that can support stakeholders in navigating the complex dynamics of AI adoption in healthcare settings.

### Ethics

In the Netherlands, ethical approval for research is required if the study falls under the scope of the Dutch Medical Research involving Human Subjects Act (In Dutch: Wet medisch-wetenschappelijk onderzoek met mensen, WMO) [28,29]. Research is subjected to this Act if it meets two cumulative criteria: 1) The research qualifies as medical-scientific research, and 2) it involves subjecting participants to procedures or imposing specific rules of behaviour on them.

As our study did not meet these criteria, approval was not needed according to Article 1b of the Dutch Medical Research in Human Subjects Act [28]. Notwithstanding, all the participants were fully informed about the study objectives, procedures, and their rights. FGIs were recorded with the verbal consent of participants, and all data were processed and stored anonymously. Participants were explicitly informed that their participation was voluntary and that they could withdraw from the study at any time without any consequences. No financial incentives or other forms of compensation were provided to participants.

## Results

### Survey

**Fig 1:**
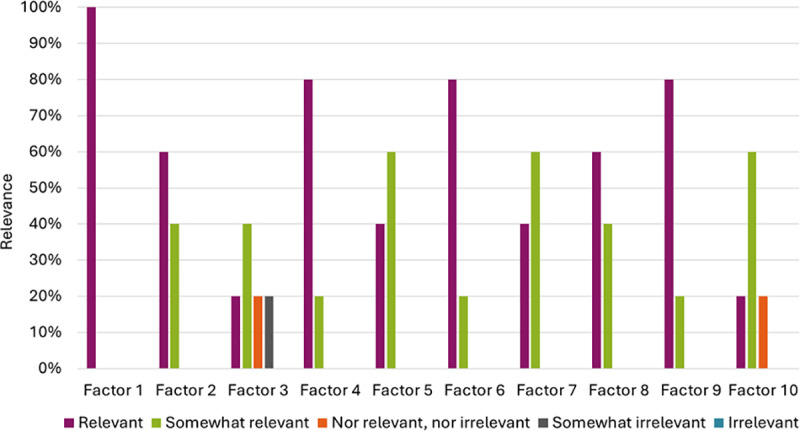
Percentage of experts rating the relevance of factors related to the societal aspects of implementation of AI technologies in smart hospitals.

Five out of seven work package leaders participated in the survey, with no reasons provided for the two who did not respond. Of the 10 factors proposed, 9 were considered relevant (90%). One factor, managing digital healthcare supply chains for the successful implementation of AI technologies in smart hospitals (factor 3), was deemed irrelevant. Fig 1 illustrates the relevance of each factor based on response categories. All factors, except factor 3, were included in the topic guide for FGIs. Additional comments were provided for factor 1 (digital integration and interconnectedness of the healthcare ecosystem), stating that there are multiple ways of integrating technologies, such as live connection or batch integration. Comments were also provided for factor 2 (utilization of big data and analytics), emphasizing that AI should be continually learning. Regarding factor 10 (ethical aspects), the comment highlighted that the most important ethical issue is ensuring the best possible patient care, even though it is not explicitly mentioned. These points have been included for discussion in the FGIs.

### Focus group interviews

A total of 11 participants took place in the FGIs, divided among four FGIs. Participants were researchers/technology developers (n = 4), HCPs and researchers/technology developers (n = 4), an implementation manager/technology developer (n = 1), an ethicist (n = 1), and a patient representative (n = 1).

### Factors

Below, the factors from the FGIs are elaborated upon (please note that the order of presentation differs from the original sequence). During the FGIs, an overlap was observed between the factors ‘digital integration and interconnectedness of the healthcare ecosystem’ and ‘utilization of big data and analytics’. Similarly, an overlap was noted between the factors’strategies for promoting the use of AI technologies’ and’promotion of a culture for the use of AI technologies’. These factors have been combined in the findings as reported below.

#### Regulatory aspects of AI technologies in medicine.

Participants mentioned that a challenging aspect is the absence of clear, AI-specific guidelines in existing regulations, such as those from the European Medicines Agency (EMA). They noted that this regulatory gap, coupled with the lack of concrete procedural guidance, creates uncertainty in the implementation of AI technologies. The European AI Act, while a step in the right direction, also seems to suffer from a lack of specificity. Moreover, the use of disconnected information technology (IT) systems across hospitals is considered a major obstacle for data-driven system development. Harmonizing regulations within hospitals and across European Union (EU) Member States was deemed essential for successful AI technology introduction and obtaining requisite amounts data of AI learning.

*“I think there is not much clarity yet to when you can share data for AI development. And I think some physicians might not be that comfortable with just sharing data on a large scale. But it’s not always up to them, of course, but it is very hospital specific.” (P1)*

Participants viewed the current period of innovation and advancement of AI as an opportunity for developers and researchers. Due to the high level of uncertainty and quick pace of innovation, the direction and clarity of regulation surrounding (decentralized) AI technologies can be influenced by the field. They recommended proactively addressing ethical, logistical, and regulatory challenges associated with AI. Central to this effort is the recognition that AI is becoming increasingly prevalent and emphasis on the need to manage these issues effectively.

#### Digital integration and interconnectedness of the healthcare ecosystem.

Digital interconnectedness within the healthcare system, facilitating data sharing among stakeholders, plays a pivotal role in the success of AI implementation in smart hospitals.

The following sections elaborate on the various aspects of this interconnectedness from different perspectives micro, meso, and macro-level.

#### Macro-level (system level).

Since most EU healthcare systems are still transitioning from paper-based to digital systems and lack electronic patient files, participants felt that adopting AI based on integrated systems as a distant prospect. Differences in both electronic systems used and protocols for handling of sensitive patient data hinder interconnectedness and data sharing. Integration is however needed for personalized medicine, which requires extensive, standardized data gathering.

*“Basically, for personalized medicine you need to generate more data than what we’re currently doing. Everybody would have to generate the same data with platforms that are so similar that you can actually lump all the data together.” (P2)*

#### Meso-level (organizational level).

Participants believed that comfort with traditional, paper-based methods hinders progress towards digital integration. Inconsistencies in agreements between systems, such as interpretation of when data is (pseudo)anonymized, pose challenges. Uncertainty about when data can be shared for AI development can also be a barrier, as some HCPs feel they have insufficient knowledge on the General Data Protection Regulation (GDPR) to engage in large-scale data sharing initiatives.

#### Micro-level (individual level).

A recommendation made was to carefully consider the desirability of sharing specific data in an interconnected manner before initiating a project. Rather than sharing all available data, it was advised to define the type of data needed and connections that need to be made within a hospital.

#### Ethical aspects of AI technologies in medicine.

Concerns include the ownership and privacy of genetic and cellular data, particularly when used for treatment. Participants also worried about access to personal data. They stressed the importance of informed consent for data use in personalized treatments and registries and emphasized that posthumous data use consent should not be assumed.

A major concern was the lack of transparency in AI algorithms, which leads to misunderstanding and mistrust. This also raises security concerns due to the risk of not being aware of potential hacking or manipulation. The dynamic, self-learning nature of some AI complicates understanding its decision-making process, highlighting the need for developing AI systems that provide reasoning behind their decisions or prediction, allowing for intellectual oversight by humans. Participants also worried about data breaches or cyber-attacks that could affect treatment safety and continuity, underscoring the necessity of contingency plans. Further, AI has the potential to exacerbate existing health inequalities, particularly when it is trained on data from majority populations but applied across diverse populations, including underrepresented minorities.

*“Regarding algorithmic fairness and bias there is a healthcare-system wide deficiency. Clinical trials, dose escalations for drugs, are all adjusted to the middle-aged average white male.” (P3)*

Participants outlined several *conditions* for ethical implementation of AI technology. They emphasized that ethical considerations should be interwoven in the design phase of projects to avoid biases and ensure fairness, especially for underserved populations. Access to diverse data is crucial for algorithmic fairness. Furthermore, the recognition and understanding of potential biases in AI systems by different stakeholder groups can help mitigate them, making education on ethical aspects vital.

It was agreed that currently, an ethically acceptable role of AI is to serve as a support tool for decision-making. Critical medical decisions should remain the responsibility of HCPs. Ensuring human oversight helps maintain trust from HCPs and patients.

Ethical issues arising from AI development and utilization often go unaddressed. Participants promoted the view of ethics not as a standalone concept or “checkbox”, but rather as an integral part of AI development and implementation.

#### Development and promotion of strategies for adopting an AI culture.

Creating a shared culture and vision is needed for stakeholder cooperation and ultimately AI adoption. Strategies must explain and promote AI innovation amongst all end-users. Engaging stakeholders and ensuring HCPs embrace automation are key. Many organizations lack a cohesive AI culture due to limited experience, leading to extreme optimism or scepticism. Demonstrating an AI’s tangible benefits can build a unified culture and foster acceptance for successful integration.

To effectively promote AI adoption, organizations should adopt a gradual approach, incorporating evaluation points throughout implementation and development. This allows for careful assessment at each step to ensure alignment with initial goals.

Participants highlighted the need for better accessibility of information. While scientific papers contribute to understanding, they often do not reach the wider public. Thus, translating complex information into accessible formats is an important mean to increase awareness for -and a pragmatic view on- AI technologies.

Participants noted that meetings can be instrumental in bringing together stakeholders to address resistance to change. By initiating discussions and promoting collaboration, meetings can address concerns and facilitate in finding common ground.

#### Leadership in healthcare innovation.

It was believed that traditionally, a healthcare leader aligns efforts amongst various HCPs. However, participants emphasized that implementing AI technology in healthcare requires collaboration among additional stakeholders, including hospital management and IT staff. Due to the complexity of the healthcare ecosystem, no single leader can oversee the entire network effectively. Thus, successful implementation depends on a collaborative effort.

HCPs among the participants noted that while a flat hierarchy is desirable, it is not yet realistic. They expressed concerns that HCPs might be held solely responsible for AI-assisted healthcare outcomes due to the absence of a specific role for this responsibility. This responsibility is not something that most HCPs are willing to take up, according to the sample in our FGI’s, certainly not without the technology knowledge needed to be responsible.

Successful adoption of AI technology depends on effective communication and collaboration among stakeholders. Encouraging HCPs to talk to each other and understand the problems they face can facilitate the successful implementation of AI systems.

*“When introducing AI based systems into a clinical environment, you have to do a lot of teaching in the beginning and answer a lot of questions and accompany every first step in the usage. Because otherwise it will fail, even if it’s a good system.” (P4)*

#### Stakeholders’ skills.

To maximize effectiveness of AI technology, continuous de-, re-, and up-skilling of those applying the system is important.

Training HCPs in medical technologies is essential for implementation, but many HCPs already feel overwhelmed. Learning new skills and adapting to new technologies require time that may not be readily available. A transition to digitalization may require additional personnel, like clinical technologists, who have expertise in both medicine and technology.

Participants outlined several conditions for successfully integrating AI in HCPs’ skills. They emphasized AI optimizing or simplifying processes rather than replacing human roles. Additionally, (clinical) studies should be conducted to assess changes in HCPs’ skills and the impact of new technology on patient care. Concerns were raised about HCPs deskilling due to AI introduction. Participants emphasized the need for training and support to mitigate skill loss and ensure new skill development. Additionally, it is important to consider that if many different AIs are introduced, users may struggle to develop the necessary skills to use all of them effectively.

FGI results showed that not only HCPs but also patients need education for successful AI implementation. Patients often lack awareness about AI, its uses, benefits, and safeguards. To bridge this gap, patient education initiatives should provide clear and accessible information about AI and its implications for healthcare.

Many tools currently lack clear evidence of clinical benefits, making it hard to convince users to adopt them. Demonstrating clear benefits, such as improved task efficiency, speed, overall performance, or user confidence, is key to gaining user support. Participants noted that multiple intermediaries between decision-makers and end-users can lead to information loss and reduced feelings of ownership. This was identified as a cause of resistance to new technologies. Overcoming this requires direct communication and stakeholder involvement in decision-making. It also requires gradually introducing IT solutions and involving individuals in the change process. Flat hierarchies and inclusive approaches can help mitigate resistance and facilitate smoother transitions.

#### Adoption of new business models.

Adequate financial conditions must be in place for the successful implementation of an AI manufacturing platform. Traditional business models may not be sufficient to capture the rapidly evolving AI technologies, which often have value-driven outcomes. Participants expressed concerns about treating medicine as a revenue-generating market, a sentiment echoed by the broader public, especially given the high prices of many drugs. They emphasized that new business models should prioritize ensuring patient access to innovative treatments.

Participants discussed exploring alternative business models in AI medicine manufacturing, such as subscription models, which reduce upfront costs by offering recurring licensing fees. Ethical considerations about data ownership and commercialization were also highlighted. Questions arise about who holds the authority to sell or transfer data for use in AI tools and their training. Additionally, scalability must be considered, requiring preparation and infrastructure development to ensure business models can handle increased demand and maintain effectiveness on a larger scale.

### Societal guide

**Fig 2:**
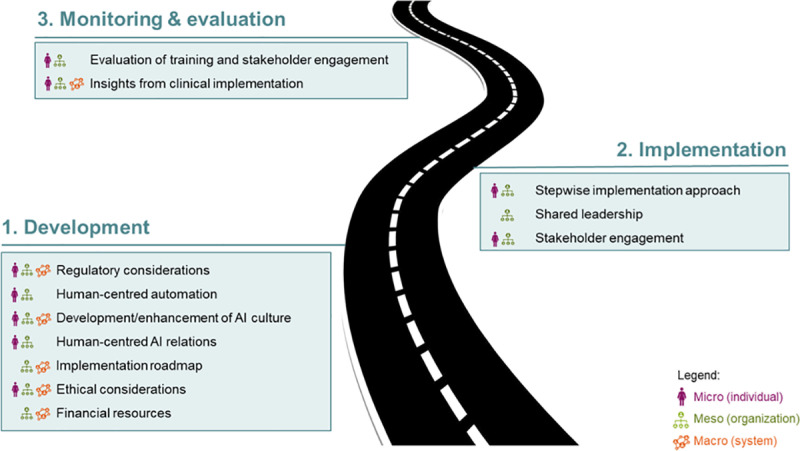
Societal guide for implementation of AI-technologies in (smart) hospitals.

In Fig 2 the societal guide is shown, which serves as a comprehensive roadmap, outlining key principles and considerations for guiding the implementation of AI-driven technologies in healthcare settings. Table 1 below elaborates on the items displayed in the roadmap. Together, they consider the dynamic landscape of technology, the (evolving) perspective of stakeholders, and (the changing) ethical and legal considerations. The guide is structured into three phases of AI innovation: 1) development; 2) implementation; 3) monitoring and evaluation. It outlines factors to consider at each phase and identifies the levels, on which these considerations come into play -micro (individual), meso (organizational) and/or macro (system)-level. Successful implementation requires a holistic view. Not only can changes or developments in one phase significantly influence the others, implementing AI into healthcare systems effectively requires a multi-level understanding. Not only should factors on each level be addressed, but it is also important to understand how individual, organizational, and systemic factors interact to prepare for societal integration of AI. S3 File gives an example of how considerations on different levels influence each other.

**Table 1:** Elaboration of factors in the societal guide for implementation of AI-technologies in (smart) hospitals, divided into micro, meso and macro levels over the developmental-, implementation- and monitor & evaluation-phase.

	Micro level (individual)	Meso level (organisation)	Macro level (system)
**Phase 1: Development**			
**Regulatory considerations**		• Implement processes for obtaining informed consent from patients and ensuring transparency regarding how their data is used. • Facilitate discussion about resistance to and concerns around implementation (e.g., regarding digitalization, data protection, cybersecurity risks). • Develop a stepwise approach for digitalization. • Develop contingency plans for data breaches and cyber-attacks. • Work towards electronic patient dossiers. • Ensure compliance with GDPR, Medical Device Regulation, and upcoming European Commission AI Act. • Deal with inconsistencies in definitions and interpretations; define terms like “anonymized data” and “pseudonymization”. • Engage with regulatory bodies to influence AI manufacturing regulations and ensure clarity. • Create a data infrastructure to support integration.	• Consider the needs from the field around regulatory frameworks. • Develop policies and regulations for an interconnected IT infrastructure, data management, and security aligned with international standards. • Emphasize disciplined standardized data entry practices. • Harmonize regulations within hospitals and across EU states for AI technology implementation.
**Human-centred automation**	• Develop explainable AI systems. • Offer transparent and clear explanations of AI functionality, including its processes, decision-making basis, and error-handling procedures. • Utilize an accessible (graphical) format/interface to present AI interventions. This interface should display decision confidence and outline steps leading to each decision.	• Standardize AI interface development to promote transparency and explainability.	
	**Micro level (individual)**	**Meso level (organisation)**	**Macro level (system)**
**Development/ enhancement of AI culture**	• Collaborate to develop training on the use of AI technologies for HCPs and technicians. • Consider workflow adjustments for HCPs and technicians in advance. Let stakeholders reflect on anticipated versus desired role changes. • Increase awareness and understanding of AI innovation and its healthcare applications. • Promote a (more) pragmatic attitude to AI implementation, acknowledging challenges in forming opinions due to its novelty and limited clinical implementation data.	• Establish an AI-oriented culture by: • Engaging all stakeholders and encouraging interdisciplinary collaboration and a flat hierarchy; • Scheduling regular stakeholder meetings; • Collaboratively defining goals and milestones to be achieved before, during, and after implementation; • Co-create education and training materials tailored relevant stakeholders’ understanding; • Developing patient education (initiatives) to enhance understanding of an AI technology’s risks, benefits, and healthcare implications.	• Encourage innovative environments. It is important for policymakers and regulators to facilitate development and harmonization within the medical technology sector. This helps cultivate environments that are supportive of AI innovation.
**Human-centred AI relations**	• Provide education as HCPs to patients. • Ensure accessibility and comprehensiveness of education materials. • Recognize different levels of AI understanding among patients and tailor discussions accordingly. • Build trust by being transparent about AI’s role in healthcare, emphasizing that decisions remain in the hands of human-beings.	• Determine responsible and accountable parties for AI technology outcomes; technology alone cannot bear responsibility. Alternatively, a shared responsibility framework can be constructed to ensure accountability. • Enhance public awareness and general understanding. This is important for building trust, promoting informed decision-making, and encouraging active engagement in AI technology discussions.	• Develop regulations on liability in case of AI technology malfunctioning in medical care.
	**Micro level (individual)**	**Meso level (organisation)**	**Macro level (system)**
**Implementation roadmap**		• Adapt the procedural guidance from regulatory bodies to align with own organizational needs and specific AI technology. • Develop an implementation roadmap outlining training opportunities for stakeholders involved in utilizing the AI technology.	• Develop concrete procedural guidance specific to AI-related instructions.
**Ethical considerations**	• Integrate ethical and privacy considerations into algorithm development and training modules. • Adress questions concerning data authority proactively. • Promote understanding of patient autonomy and the importance of informed consent in the use of AI technologies in patients. • Consider ethical implications of data ownership and commercialization.	• Establish clear guidelines and protocols for data collection, storage, and usage within the smart hospital ecosystem. • Integrate privacy considerations throughout AI technology development, including algorithm selection and data analysis techniques. • Emphasize ethical consent and privacy, particularly in data sharing for AI development.	• Develop standardized ethical frameworks tailored to various stakeholder groups. • Address issues related to sensitive patient data management, privacy, algorithmic fairness, transparency, and explainability.
**Financial resources**		• Explore innovative business models like subscription models or open development competitions. • Adopt a patient-centric approach that prioritizes treatment accessibility over profit-driven motives. • Draw insights from (less) successful examples of business model execution, particularly in the context of ATMPs. • Consider scalability in the development of new business models, ensuring infrastructure and planning align with potential scaling requirements.	• Address barriers to accessing (venture) capital for funding new AI medicine manufacturing business models. • Allocate resources to support information integration and accessibility within (smart) hospitals.
	**Micro level (individual)**	**Meso level (organisation)**	**Macro level (system)**
**Phase 2: Implementation**			
**Stepwise implementation approach**	• Implement the AI technology in a stepwise manner, preferably, following an implementation roadmap.	• Adopt a gradual approach to AI technology implementation, allowing for thorough consideration of each stage and incorporating evaluation points throughout the process.	
**Shared leadership**		• Establish clear lines of responsibility and (shared) leadership structures to facilitate effective decision-making.	
**Stakeholder engagement**	• Prevent stakeholders from feeling overwhelmed by scheduling to time get used to the training modules and technology. • Schedule time for stakeholders to reflect on anticipated and actual role changes during implementation.	• Ensure inclusivity and engagement throughout the implementation process, ensuring alignment towards a shared vision after implementation.	
**Phase 3: Monitoring and evaluation**			
**Evaluation of training and stakeholder engagement**	• Evaluate the applicability of the training modules with relevant stakeholders. • Monitor HCPs’ skills proficiency and adapt training modules if necessary.	• Organize regular meetings to (re)evaluate (interim) goals and steps. • Check whether there is (still) resistance and address any resistance by involving these stakeholders. • Encourage discussions and collaboration to find common ground.	
**Insights from clinical implementation**	• Collect and analyse clinical implementation data. • Investigate workflow changes and their impact on patient care and HCP satisfaction.	• Develop an evaluation-based checklist for future use. • Disseminate good practices and lessons learned.	• Promote knowledge sharing and good practices by compiling an overview. • Identify locations of good practices and establish connections between them. • Improve accessibility of information about AI technologies for the broader population.

## Discussion

### Principal findings

This study sought to identify societal factors influencing the implementation of AI technology according to different stakeholder groups and to explore how these factors play a role within (smart) hospitals. Through a survey and focus group interviews, we discussed the facilitators, barriers, possibilities, and preconditions of selected factors. Based on the insights gathered from these discussions, a societal guide was developed. This guide serves as a comprehensive roadmap, outlining key principles and considerations for guiding the implementation of AI-driven technologies in healthcare settings. It considers the dynamic landscape of technology, the evolving perspective of stakeholders, and the changing ethical and legal considerations.

It was found that while much knowledge exists on different factors concerning AI in (smart) hospitals, this knowledge is often siloed. This knowledge must be integrated across stakeholders across different phases of AI implementation to adequately prepare for the deployment of AI technologies. This ensures that insights gained in each phase can inform and enhance the subsequent phases

Most of the insights from FGI participants were found to belong to phase 1, development. This underscores the need for extensive preparation on all levels before entering the implementation phase. Furthermore, recognizing the interdependence among factors such as digital systems, regulatory requirements, ethical considerations, and stakeholder involvement is key in forming a robust implementation strategy.

Focus group discussions highlighted that some issues need to be addressed at different levels to be resolved effectively. Therefore, the societal guide is meant to serve various purposes for different groups. Regulatory bodies and policymakers can make use of it to create effective guidelines that ensure responsible and ethical use of AI technologies (macro level). Healthcare institutions and manufacturers can follow its recommendations to facilitate the introduction of the AI technology to stakeholders (meso level), thereby addressing potential resistance and ensuring that all parties are aligned in their objectives from the beginning. Together, this can create an inductive environment for AI implementation at the micro level. Researchers, innovators and HCPs can subsequently use the guide to refine their strategies, considering societal expectations (micro level). Use of one framework or guide can also establish a more uniform approach towards AI introduction, as there currently seems to be a lack of direction. In addition to the factors discussed, long-term monitoring and updating of AI technologies in healthcare is essential to maintain their effectiveness. As healthcare systems evolve, AI must be continually evaluated and refined to adapt to new medical knowledge, regulations, and ethical concerns. A strong monitoring framework will help identify biases, errors, or discrepancies, ensuring AI remains aligned with healthcare needs. Collaboration among stakeholders—regulatory bodies, healthcare providers, and AI developers—is necessary to establish ongoing evaluation and updates, ensuring AI accuracy and reliability.

### Comparison with prior work

Comparing our findings with existing literature on the societal aspects of AI implementation in both industry and healthcare shows that research in this area is emerging. While our analysis provides insights into current perspectives, it is anticipated that these factors will evolve, and further clarity will emerge regarding specific societal factors influencing the adoption and impact of AI implementation in (smart) hospitals.

Participants in the FGIs echoed many concerns highlighted in existing literature regarding AI’s challenges in healthcare, such as the potential for increased unemployment due to automation [5,7–9,11,30], risks related to patient data privacy [7,9,11,30], cybersecurity [30], and individual autonomy [9,10,12,30]. Similarly, our study replicates the fear that AI could worsen inequalities by primarily benefiting those who already have access to advanced healthcare, while leaving marginalized communities further behind [5,7–11]. Moreover, AI algorithms are likely to be trained on data reflecting existing biases in society. Output of such algorithms might result in unfair and discriminatory practices against certain groups, and users of AI should be (made) aware of that [7,12]. One of the significant challenges associated with AI is the ‘black box’ problem, where the decision-making processes of the algorithm are not transparent or understandable to users. This lack of transparency can make it difficult to identify and address potential biases or errors within the system. To mitigate these issues, there is a growing emphasis on developing ‘explainable AI,’ where the inner workings of AI models are made more transparent and interpretable for users [9,10,30,31]. An additional ongoing debate is the question of accountability and responsibility – who is held accountable for the actions and decisions of AI systems; can you be responsible if you do not understand how an AI model got to its outputs? [5,9,11]. Recognizing that many concerns need to be addressed, AI is also recognized for its potential to enhance productivity, accessibility, and affordability of healthcare [5–9]. Both the societal guide in this study and prior studies emphasize the importance of addressing ethical concerns, such as algorithmic fairness and transparency, early on to build public trust in AI [9,11,12,32]. In line with existing research, FGI participants stressed the need for improving AI literacy across all sectors and securing international cooperation to enhance AI’s perception and ensure its safe implementation [9,33]. Clear regulations on AI should be not only be made by and be readable to policymakers but also understandable to the general public, doctors, and patients [9,10].

However, FGI participants did not touch on some insights from other literature. For example, research suggests that companies can gain a competitive edge by proactively addressing ethical issues through corporate social responsibility (CSR), which not only contributes to a safer society but also can strengthen brand reputation and customer trust [30]. Additionally, another paper proposes a novel approach to managing AI risks, advocating for society to be prepared to address unforeseen negative effects of AI [33]. The authors of this paper point towards educators, governments and journalists to continually make the general public, industry leaders, and decision-makers aware of what advanced AI systems are capable of. Amongst others, their recommendations include implementing staged releases of AI systems to give society more time to implement adaptations, sanctioning harmful uses of AI, and funding research to measure and predict AI risks [33]. Although AI implementation has been widely studied, less attention has been given to its long-term integration and monitoring in healthcare. Continuous oversight is crucial to address emerging issues and ensure safety and efficacy [19,34]. Patient acceptance is equally vital, influenced by trust, reliability, and understanding of AI [35,36]. Without this, even the most advanced AI systems may fail to integrate into clinical practice.

### Strengths and limitations

To the best of our knowledge, this study is the first to investigate societal factors related to AI-driven technologies and combine them into a societal guide. This research addresses a gap by exploring the influence of societal dynamics on the implementation of AI in healthcare. When interpreting the results, it is important to consider the following limitations. The survey results showed that participants found the management of digital healthcare supply chains (factor 3) irrelevant. However, the reasons for this were unclear, as the survey did not explore why certain aspects were deemed irrelevant. Understanding this perception may help identify overlooked barriers or mismatches between current AI innovation and healthcare operational realities. Secondly, challenges arose during the recruitment process for the FGIs, primarily due to the innovative and emerging nature of AI in healthcare. The scarcity of individuals with the necessary knowledge and expertise on AI implementation presented difficulties, resulting in the absence of key stakeholders, such as representatives from regulatory affairs, nurses, and technology operators/IT professionals. Additionally, the use of convenience sampling, which drew only from the direct networks of consortium partners, likely contributed to this limitation. This sampling approach may affect the generalizability and broader applicability of the guide, as it may not reflect the views of all stakeholder groups who work with or are affected by AI in healthcare. Third, it became apparent that some participants had a tendency toward tunnel vision and did not look beyond specific barriers that directly impacted their own experiences or interests. This tendency was considered a limitation in the FGIs because it was difficult to get participants to think beyond their own concerns and consider broader perspectives.

### Recommendations

#### Research.

Further investigation is recommended on factor ‘management of digital healthcare supply chains’ (factor 3) to understand why it was considered irrelevant by participating experts. Additionally, it is important to include the perspectives of underrepresented stakeholder groups that were not part of the current research, such as experts from regulatory affairs, nurses and other professional groups, and technology operators, to ensure a more comprehensive perspective in the societal guide. As the success of an AI technology also depends on its ability to enhance the patient experience and prioritize patient-centricity, future research should explore how AI technologies impact various dimensions of patient experience. Considering that underserved healthcare systems may encounter substantial challenges with digital integration and ethical frameworks, further research should explore the specific needs and constraints of these settings in AI implementation. To deepen insight and enhance the robustness of future findings, a mixed-methods approach is recommended. While this study offers qualitative insights into stakeholder perspectives, quantitative methods could provide broader generalizability and highlight patterns across different settings. For instance, surveys could be conducted to assess the prevalence of identified barriers and facilitators across stakeholder groups or regions. Structured questionnaires could also measure levels of AI readiness, digital literacy, or trust in AI among healthcare professionals and patients. Such efforts will help create a more inclusive and effective societal guide that reflects a wide range of healthcare environments.

#### Practice.

Several actions were recommended by the FGIs participants to ensure the successful implementation of AI technologies. One of them was a comprehensive approach that integrates various elements – such as regulatory frameworks, ethical considerations, digital infrastructure, human factors, and financial resources – is essential. Equally important is fostering a culture of knowledge sharing within the stakeholder community. This involves not only sharing good practices, but also learning from failures and challenges. By openly sharing both successful examples and lessons learned, stakeholders can gain valuable insights into what works well and what pitfalls to avoid. Additionally, transparency in sharing both successful examples and lessons learned promotes collective learning, which in turn can enhance the effectiveness and sustainability of AI technology integration in healthcare. When using the societal guide, it is important to consider the specific socio-cultural, economic, and regulatory context of each country. Tailoring the guide enables stakeholders to effectively address local barriers and enhance the impact of AI technology integration in medicine. For example, Europe faces challenges in accessing venture capital for funding new business models compared to the US and Asia, which hinders innovation and development in the medical technology sector. To address these funding challenges, innovation-friendly environments should be fostered in Europe. A tool that can guide development and scale-up of AI within existing laws and regulations is, for example, the’innovation funnel for valuable AI in healthcare’, developed by the Dutch Healthcare institute. This funnel considers five domains (value, application, ethics, technology, responsibility), which are key in the innovation process [37].

Recommendations for patient education on AI include organizing webinars covering basic AI concepts and applications in healthcare, such as smart hospitals, diagnosis, prognosis, and personalized medicine. These webinars could feature expert panels and include interactive Q&A sessions. Additionally, providing information in diverse formats, such as patient leaflets and animated videos, ensures broader accessibility and understanding among patients of different backgrounds and age groups.

When introducing new AI systems, it is recommended to proceed in small steps. For example, initially AI can serve as a second pair of eyes for HCPs, which gradually transitions to interpreting AI generated judgments, and ultimately builds towards trust in the technology for decision-making. Furthermore, HCPs will need time to reflect on the changing roles and responsibilities resulting from the integration of AI into healthcare practice. Another recommendation is to implement visualizing tools that illustrate how decisions are derived from AI models. Measures to verify the quality of AI generated results could be incorporated, such as confidence numbers or scales that represent the confidence of an AI in its decisions.

Lastly, establishing a framework to guide both developers and users of AI systems was strongly recommended. This framework should incorporate diverse perspectives to ensure stakeholder consensus and provide a roadmap for daily activities. Currently, such a framework does not exist, indicating room for development in this area.

## Conclusion

The findings of this study highlight the uncertainty surrounding the future use of AI technologies within (smart) hospitals. As this field is still in its early stages, there are limited established methodologies, as well as (regulatory and ethical) frameworks, to guide its implementation. Our societal guide addresses these gaps by focussing not only on ethical and regulatory considerations but also on important human-centred factors for AI implementation in healthcare. Engaging various stakeholders across all phases –the development, implementation, monitoring and evaluation phase– is key for fostering a collaborative approach. Recognizing the interdependence of ethical, regulatory, digital and human-centred factors, and understanding their collective impact on successful implementation, is essential. The societal guide should be seen as an initial step towards establishing a comprehensive framework for AI technologies in medicine. It is anticipated that the guide will be refined and enhanced over time, adapting to new insights and developments. By using this guide, stakeholders can better plan their implementation approach, thereby increasing the chances of success, and ensure that the strategy is tailored to the specific needs and challenges of a healthcare setting.

## Supporting information

S1 File Survey. (PDF)

S2 File Topic guide FGIs. (PDF)

S3 File Venn diagram of factors at different levels in the development phase of AI implementation. (PDF)
