# Supplementary material for: Societal factors influencing the implementation of AI-driven technologies in (smart) hospitals
Source: PLoS One. 2025 Jun 12;20(6):e0325718. doi: 10.1371/journal.pone.0325718 (PMC12161522; doi:10.1371/journal.pone.0325718)
Supplement: S1 File — (PDF) [file pone.0325718.s001.pdf]

Supplementary file 1 - Survey

Factor 1: Digital integration and interconnectedness in the healthcare ecosystem

In the literature it is reported that for the success of the (implementation of) AI technologies, a highly integrated and interconnected healthcare ecosystem is crucial. This requires digital connectivity between technologies used in this healthcare ecosystem by government agencies, hospitals, health organizations, insurance companies, pharmacies, and others, facilitating smooth communication among them. Working together, healthcare professionals, policymakers, tech experts, and patients can create a unified and coordinated way to implement new technologies. To prevent problems, standardized practices are crucial for all involved parties. Data sharing supports effective collaboration, but patient privacy must be upheld. The technology infrastructure also matters in enabling connectivity among stakeholders, but it differs between countries, some having more advanced systems than others.

1. How relevant is digital integration and interconnectedness of the healthcare ecosystem with regard to successful implementation of AI technologies (e.g. AIDPATH) in smart hospitals?

- o Relevant
- o Somewhat relevant
- o Nor relevant, nor irrelevant
- o Somewhat irrelevant
- o Irrelevant

2. Do you have any additional comments and/or suggestion regarding this assessment?

Factor 2: Utilization of big data and analytics

The successful implementation an innovative AI technology relies on leveraging big data analytics in both patient care and hospital operations management. These big data analytics will be used for both optimizing manufacturing processes, improving capacity management, and scheduling both platform tasks and patient flow through the process. A challenge here is ensuring data from devices is securely stored while simultaneously prioritizing patient privacy.

3. How relevant is using big data and analytics with regard to successful implementation of AI technologies (e.g. AIDPATH) in smart hospitals?

- Relevant
- Somewhat relevant
- Nor relevant, nor irrelevant
- Somewhat irrelevant
- Irrelevant

4. Do you have any additional comments and/or suggestion regarding this assessment?

Factor 3: Management of digital healthcare supply chains

AI technologies can drive improvements to traditional supply chains. Digital healthcare supply chains use technology and data analysis to improve product and service delivery, thus boosting efficiency. They also provide more insights in the supply chain. This not only cuts costs but also can improve patient flows. Adopting such technologies needs healthcare organizations to invest in tech, data skills, and talent to be able to utilize these data analytics and manage supply chains effectively.

5. How relevant is managing digital healthcare supply chains with regard to successful implementation of AI technologies (e.g. AIDPATH) in smart hospitals?

- Relevant
- Somewhat relevant
- Nor relevant, nor irrelevant
- Somewhat irrelevant
- Irrelevant

6. Do you have any additional comments and/or suggestion regarding this assessment?

Factor 4: Strategies for promoting the use of AI technologies

Literature suggests that for successful implementation it is crucial to create strategies that promote adoption of AI technologies by the end-users of the product of the AI technology. (Think about the healthcare workers administering the product, the patients receiving the product, the hospitals applying the technology to create the product). Stakeholders play a pivotal role in formulating and implementing these strategies. Successful implementation also relies on how much healthcare professionals embrace the automation. By automating tasks for better productivity, healthcare employees and technicians can be freed up for e.g. direct patient care, critical thinking, and important decisions.

7. How relevant are strategies for promoting the use of AI technologies with regard to successful implementation of AI technologies (e.g. AIDPATH) in smart hospitals?

- o Relevant
- o Somewhat relevant
- o Nor relevant, nor irrelevant
- o Somewhat irrelevant
- o Irrelevant

8. Do you have any additional comments and/or suggestion regarding this assessment?

Factor 5: Promotion of a culture for the use of AI technologies

For the successful implementation of the platform, it is crucial to keep into account the shared values and beliefs among all healthcare stakeholders regarding the use of AI technologies. Social aspects need to be addressed alongside technological advancements.

9. How relevant is promoting a culture for the use of AI technologies with regard to successful implementation of AI technologies (e.g. AIDPATH) in smart hospitals?

- o Relevant
- o Somewhat relevant
- o Nor relevant, nor irrelevant
- o Somewhat irrelevant
- o Irrelevant

10. Do you have any additional comments and/or suggestion regarding this assessment?

Factor 6: Leadership in healthcare innovation

Health leadership is the capacity to recognize priorities, offer strategic guidance, guide stakeholders, and build sector-wide commitment for implementation. A smart hospital needs a visionary leader to effectively utilize the platform. This leader guides stakeholders, encourages platform use, and directs the healthcare system toward successful adoption of technology.

11. How relevant is healthcare leadership with regard to successful implementation of AI technologies (e.g. AIDPATH) in smart hospitals?

- Relevant
- Somewhat relevant
- Nor relevant, nor irrelevant
- Somewhat irrelevant
- Irrelevant

12. Do you have any additional comments and/or suggestion regarding this assessment?

Factor 7: Development of skills among healthcare professionals

To achieve success with the platform, continuous de-skilling, re-skilling, and up-skilling of involved (healthcare) workers is important. Training and equipping healthcare professionals with the necessary expertise in utilizing platform outputs and technologies will maximize its benefits and effectiveness. However, the global shortage of healthcare workers, particularly specialists and nurses, poses challenges, leaving limited time for training in new technologies.

13. How relevant are healthcare professionals' skills with regard to successful implementation of AI technologies (e.g. AIDPATH) in smart hospitals?

- Relevant
- Somewhat relevant
- Nor relevant, nor irrelevant
- Somewhat irrelevant

- Irrelevant

14. Do you have any additional comments and/or suggestion regarding this assessment?

Factor 8. Adoption of new business models

Inadequate financial support structures could hinder the uptake of AI technologies according to literature. Nevertheless, the successful implementation of AI manufacturing platform opens up possibilities for discovering improved business models that capitalize on digital technologies to generate revenue. You can think of a platform regardless of the technology or device providers involved. There are challenges to address, such as convincing
stakeholders of the benefits and dealing with the high cost of technology. Despite these issues, embracing novel approaches like this lets healthcare groups explore new revenue streams and excel in the digital age.

15. How relevant is adoption of new business models with regard to successful implementation of AI technologies (e.g. AIDPATH) in smart hospitals?

- Relevant
- Somewhat relevant
- Nor relevant, nor irrelevant
- Somewhat irrelevant
- Irrelevant

16. Do you have any additional comments and/or suggestion regarding this assessment?

Factor 9: Regulatory aspects of AI manufacturing platforms

The AI manufacturing platform and the cells it produces will be subject to a variety of regulations. Examples include regulatory pathways for bringing advanced therapy medicinal products (ATMPs) from clinical trials to market authorization [1], data protection measures for health-related personal data under the General Data Protection Regulation (GDPR) [1], the Medical Device Regulation [2] and the upcoming European Commission AI act [3]. Additionally, point-of-care manufacturing in a large number of smart hospitals will bring about additional regulatory challenges, as regulatory agencies need to learn to ensure the continued safety, efficacy, and

quality of medicinal products manufactured outside centralized facilities [4]. Apart from complying with current regulations, preparations can be made for compliance with the future AI act and AI risk management.

17. How relevant are regulatory aspects of AI manufacturing platforms with regard to successful implementation of AI technologies (e.g. AIDPATH) in smart hospitals?

- Relevant
- Somewhat relevant
- Nor relevant, nor irrelevant
- Somewhat irrelevant
- Irrelevant

18. Do you have any additional comments and/or suggestion regarding this assessment?

Factor 10: Ethical aspects of medicinal AI technologies

Since there is no standardized regulation for AI implementation yet, ethical issues arising from development and utilisation of AI technologies remain largely unaddressed. A literature review aiming to identify the ethical issues of AI application in healthcare found that most frequently reported issues were: algorithmic fairness, preservation of human autonomy, explicability and privacy [5].

19. How relevant are ethical aspects of medicinal AI technologies with regard to successful implementation of AI technologies (e.g. AIDPATH) in smart hospitals?

- Relevant
- Somewhat relevant
- Nor relevant, nor irrelevant
- Somewhat irrelevant
- Irrelevant

20. Do you have any additional comments and/or suggestion regarding this assessment?

21. Can you think of other additional factors that are relevant for the successful implementation of AI technologies (e.g. AIDPATH) in smart hospitals?

Sources used:

[1] European CAR-T Handbook, co-promoted by the European Society for Blood and Marrow Transplantation (EBMT) and the European Hematology Association (EHA) Chapter 37. The Regulatory Framework for CAR-T Cells in Europe: Current Status and Foreseeable Changes AND Centre Qualification by Competent Authorities and Manufacturers

[2] European Medicines Agency (2023) Medical Devices. https://www.ema.europa.eu/en/humanregulatory/overview/medicaldevices#:~:text=The%20Medical%20Devices%2 0Regulation%20applies,on%20active%20implantable%20medical%20devices.

[3] European Commission (2023) A European approach to artificial intelligence. https://digitalstrategy. ec.europa.eu/en/policies/european-approach-artificial-intelligence. Visited on: 23/08/23

[4] Bicudo The UK's emerging regulatory framework for point-of-care manufacture: insights from a workshop on advanced therapies. (2021)

[5] Karimian, G., Petelos, E. & Evers, S.M.A.A. The ethical issues of the application of artificial intelligence in healthcare: a systematic scoping review. AI Ethics 2, 539–551 (2022). https://doi.org/10.1007/s43681-021-00131-7

-- THE END--
