# Supplementary material for: Societal factors influencing the implementation of AI-driven technologies in (smart) hospitals
Source: PLoS One. 2025 Jun 12;20(6):e0325718. doi: 10.1371/journal.pone.0325718 (PMC12161522; doi:10.1371/journal.pone.0325718)
Supplement: S2 File — (PDF) [file pone.0325718.s002.pdf]

# Supplementary file 2. Topic guide FGIs

**Main topics**

Factor 1: Digital integration and interconnectedness in the healthcare ecosystem;

Factor 2: Utilization of big data and analytics;

Factor 3: Developing and promoting strategies for adopting a culture for the use of AI technologies

Factor 4: Leadership in healthcare innovation;

Factor 5: Development of skills among healthcare employees;

Factor 6: Adoption of new business models.

Factor 7: Regulatory aspects of AI manufacturing platforms

Factor 8: Ethical aspects of medicinal AI technologies

<u>Questions for each factor:</u>

- What facilitators can you think of related to this factor? Can you explain why?
- What barriers can you think of related to this factor? Can you explain why?
- What possibilities can you think of related to this factor? Can you explain why?
- Which conditions must be in place concerning this factor? Can you explain why?

**Closing**

- Are there any other additional societal factors that you consider relevant for the successful implementation of AI technologies in smart hospitals, that have not been discussed today? Or would you like to share anything relevant?
