# Supplementary material for: Societal factors influencing the implementation of AI-driven technologies in (smart) hospitals
Source: PLoS One. 2025 Jun 12;20(6):e0325718. doi: 10.1371/journal.pone.0325718 (PMC12161522; doi:10.1371/journal.pone.0325718)
Supplement: S3 File — (PDF) [file pone.0325718.s003.pdf]

# Supplementary file 3. – Venn diagram of factors at different levels in the development phase of AI implementation

This Venn diagram illustrates factors at different levels - micro, meso, and macro - within the development phase. The overlapping areas represent where these levels interact, jointly creating a societal environment conducive to AI implementation in healthcare. For instance, collaboration between individuals (e.g., healthcare providers) and organizations is necessary to develop and standardize explainable AI systems. This alignment can ensure that AI applications are transparent, ethically grounded, and tailored to the needs of both users and patients.

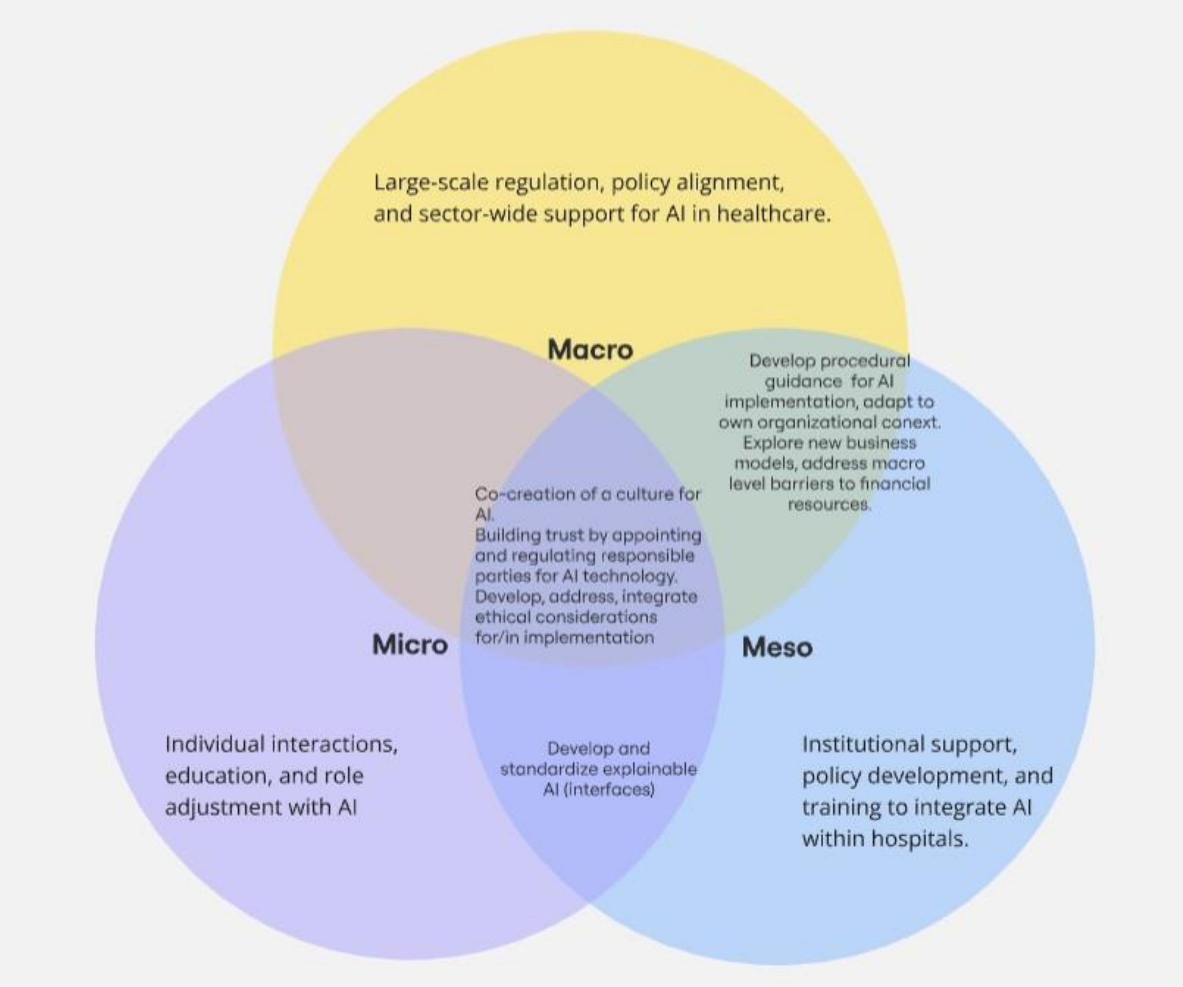


Figure 1. Considerations for societal implementation of AI in healthcare: individual (micro), organizational (meso), and systemic (macro) perspectives and their interplay
